# Brain Pathways in LIS1-Associated Lissencephaly Revealed by Diffusion MRI Tractography

**DOI:** 10.3390/brainsci13121655

**Published:** 2023-11-29

**Authors:** Alpen Ortug, Briana Valli, José Luis Alatorre Warren, Tadashi Shiohama, Andre van der Kouwe, Emi Takahashi

**Affiliations:** 1 Athinoula A. Martinos Center for Biomedical Imaging, Massachusetts General Hospital, Boston, MA 02129, USA; aortug@mgh.harvard.edu (A.O.);; 2 Department of Radiology, Harvard Medical School, Boston, MA 02115, USA; 3 Department of Behavioral Neuroscience, Northeastern University, Boston, MA 02115, USA; 4 Department of Pediatrics, Graduate School of Medicine, Chiba University, Chiba 260-8677, Japan

**Keywords:** lissencephaly, MRI, DTI, LIS1, tractography

## Abstract

Lissencephaly (LIS) is a rare neurodevelopmental disorder with severe symptoms caused by abnormal neuronal migration during cortical development. It is caused by both genetic and non-genetic factors. Despite frequent studies about the cortex, comprehensive elucidation of structural abnormalities and their effects on the white matter is limited. The main objective of this study is to analyze abnormal neuronal migration pathways and white matter fiber organization in LIS1-associated LIS using diffusion MRI (dMRI) tractography. For this purpose, slabs of brain specimens with LIS (*n* = 3) and age and sex-matched controls (*n* = 4) were scanned with 3T dMRI. Our high-resolution ex vivo dMRI successfully identified common abnormalities across the samples. The results revealed an abnormal increase in radially oriented subcortical fibers likely associated with radial migration pathways and u-fibers and a decrease in association fibers in all LIS specimens.

## 1. Introduction

Malformations of cortical development occur during the prenatal period and can be caused by various etiologies, including genetic, infectious, vascular, or metabolic factors [1]. These malformations can be caused at any stage of neuronal proliferation, migration, or postmigration cortical organization [2,3]. Classical lissencephaly (LIS), also called agyria-pachygyria, is a rare developmental disorder (around 1.2 per 100,000 births) caused by defects in neuronal migration, leading to changes in sulci, gyri, and neocortical lamination [4], and characterized by a smooth surface of the cerebral cortex [5]. Historically, LIS was divided into two groups: type I LIS (classic type) and type II LIS (cobblestone). However, because the pathophysiology of cobblestone malformations is different, type II LIS is now excluded from the list of LIS [1].

Many different types of LIS have been characterized, and an expanded classification of this disorder was recently proposed based on analysis of structural MRI data collected from 1400 patients [6]. This expanded classification considers gradient (anterior, posterior, or diffuse) and various grades of gyral malformations, including agyria (cortical areas with sulci more than 3 cm apart), pachygyria (cortical areas with sulci 1.5 to 3 cm apart), subcortical band heterotopia (abnormal layer of neurons under the normal cortex), abnormal cortical thickness (thick areas = 10–20 mm, thin areas = 5–10 mm, or variable), and the presence of non-cortical brain malformations (e.g., dysgenesis of basal ganglia, cerebellum, and brainstem).

This classification reveals a close association between phenotypic characteristics and genetic causes of these malformations. For example, patients with a predominantly posterior lissencephalic cortex [6] may be associated with mutations in the LIS1 gene [7,8] or in genes belonging to the LIS1-tubulin pathway (for review, see [9]). In contrast, patients with a predominantly thick, anterior lissencephalic cortex [6] typically have mutations in the doublecortin (DCX) gene [10,11] or actin gene isoforms (ACTB, ACTG1) [12]. In addition, the distinction between classic LIS, characterized by a thick cortex with two or four layers, and the variant form as a mild form with three layers is no longer a valid definition. As the new classification shows, different types of LIS are characterized by specific structural abnormalities (e.g., cerebellar hypoplasia, thick cortex, microcephaly) that share the absence of cortical convolutions in various regions of the cerebral cortex. Current classification considers the grade (severity) and gradient of the gyral malformation, cortical thickness, and presence of associated brain malformations [1]. LIS may be isolated or may be part of other syndromes, such as Miller–Dieker syndrome (MDS) and Walker–Warburg syndrome. Regardless, the disease itself is rare, the symptoms are highly severe, and the life expectancy is unfortunately short. 

At the cellular level, all types of LIS result from abnormal neuronal migration, which in turn leads to other abnormal developmental processes. Cortical convolutions are closely associated with a variety of neurogenic activities. These include the formation of radial glial scaffolds that aid in the radial migration of neurons [13,14]. In addition, rapid growth of white matter compared to gray matter has been observed, indicating the evolution of connectivity [15]. Other factors include the progressive growth of cortical fibers (Kostovic and Rakic 1990) and the expansion of brain volume [16,17]. Furthermore, there is a difference in the rate of thickness increase between the supragranular (I-III) and infragranular (V-VI) layers [18]. Since these processes may be impaired in LIS, in this study, we aim to analyze abnormal neuronal migration pathways and white matter fiber organization in LIS1-associated LIS compared to age-matched controls to determine which morphological differences are associated with impairment.

## 2. Materials and Methods

Slabs of formalin-fixed specimens with LIS (*n* = 3, Age: 1 year, 8 year, and 13 year) and age, sex, and regionally matched postmortem control slabs (*n* = 4) were obtained from the University of Maryland Brain and Tissue Bank (UMBTB) through the NIH NeuroBioBank (NBB) network. Cerebrum coronal brain blocks were approximately 2 cm thick. Two cerebellum specimens were sagittal blocks and approximately 2 cm thick as well. 

### 2.1. Diffusion MRI Acquisition and Analysis

As the specimens were formalin-fixed, the ex vivo imaging protocol is different from routine dMRI protocols. The primary obstacles in conducting high-resolution ex vivo human dMRI studies involve the significant decrease in T2 and diffusivity in fixed tissue [19]. To address these challenges, we extended the scan time to up to 8 h and adjusted the parameters based on the previous literature on fixed human brain MRI [20,21].

Slabs of the postmortem brains were prepared for MRI scanning by soaking them in bags containing Fomblin oil (same used as, e.g., [22]. These bags were arranged side by side and on top of each other within a container, with plastic plates dividing them. The scans were performed at the Athinoula A. Martinos Center for Biomedical Imaging. Appropriate acquisition parameters were determined by referring to the literature [20,21]. Diffusion-weighted data were acquired using a 3 Tesla SIEMENS MAGNETOM TrioTim syngo MR B17 at a 149 Hz/px bandwidth. The following parameters were used: TR = 24.84 ms, TE = 24.42 ms, flip angle = 35°, Resolution: 0.8 mm isotropic, the in-plane field of view = 144 mm × 144 mm, slice number = 104. Diffusion weighting was performed along 60 directions (diffusion gradient duration = 20 ms, bval = 1000 s/mm^2^) with 10 T2-weighted (low-B) measurements, and the total scan time was 8 h 14 min 26 s. 

TrackVis (Version 0.6.1) and the Diffusion Toolkit were used to reconstruct and visualize tractography. The FACT algorithm and 45-angle thresholds were used in the DTI model to reconstruct tractography pathways. No threshold of fractional anisotropy (FA) was used for the fiber reconstruction [23].

Regional qualitative analysis of the pathways was analyzed through these reconstructions. 

Since different slabs of brain sections were scanned together in a box, each slab was segmented using the Amira software (3D Version 2021.2, Thermo Fisher Scientific, Waltham, MA, USA) [24]. Each segmentation was used as an ROI for the corresponding specimen in Trackvis. We used interactive atlases of coronal brain anatomy (developed by the University of British Columbia and Sackler Faculty of Medicine, Tel Aviv University) to define the levels of the sections and to name the main anatomical regions [25,26]. Those main anatomical regions, such as the thalamus, basal ganglia, and deep cerebellar nuclei, were used to name and evaluate the neighboring anatomical pathways. In order to define the short and long-range fibers in the slab, we used the fiber length threshold (mm) in TrackVis.

### 2.2. Information about the Cases

#### 2.2.1. Lissencephaly

Case 1, #683, was an 8 years and 10-month-old Caucasian female who was a LIS patient, and the cause of death was complications of the disorder. She was diagnosed with LIS, agenesis of the corpus callosum, visual impairment (secondary to colobomas in both eyes), bilateral hearing loss, and severe scoliosis. The cortical ribbon was markedly thickened with a very faint white line separating the 4 mm gray superficial cortex from the markedly thickened deeper cortical band up to 2 cm in thickness. The white matter was apparently well-preserved with focal gray matter heterotopias lateral to the dilated ventricle. The basal ganglia, thalamus, and hypothalamus were somewhat abnormally disposed and configured with indistinct separation of the caudate nucleus from putamen. The corpus callosum was apparently formed but somewhat thin. The neuropathology report described a mechanism of markedly thickened gray matter sulcal dysplasia that is associated with a specific genetic defect, sometimes designated LIS1, that constitutes Miller–Dieker syndrome involving 17p13.3. The abnormalities in the patient’s eyes are of particular interest because it is a pathological process often associated with this type of brain malformation. These have been designated ‘cerebro-ocular dysplasia’ and include the ‘Walker-Warburg syndrome.’ Her medical report included severe type I LIS, and her final pathology report summed her disorders as ‘multiple congenital anomalies’. The postmortem interval was 3 h.

Case 2, #4524, was 13 years and 11-month-old Caucasian male who was a LIS patient, and the cause of death was complications of the disorder. His medical report included moderate or grade 3 LIS. His chromosome analysis showed G-banded cells, 46 XY chromosome patterns, and no visible deletion of chromosome 17. A submicroscopic deletion in the LIS critical region was detected by in situ hybridization. His final neuropathological diagnosis included type I LIS with genetic confirmation of LIS1 (Miller–Dieker) microdeletion. Accordingly, he had a thick, four-layered cerebral cortex in the retro-sylvian cerebrum-pachygyria of the brain from the frontal lobe to the Sylvian fissure and agyria in the dorsal brain, thin to non-existent corpus callosum, enlarged posterior ventricles, hypoplastic hippocampus and hypoplastic degenerating cerebellum, as well as heterotopic remnants of inferior olive in the mid medulla within the migratory track. The hippocampal formation was normally formed, and the deep gray matter structures appeared to be normal. The postmortem interval was 9 h. 

Case 3, #641, was a 1 year and 5-month-old Caucasian female who was a LIS patient, and the cause of death was complications of the disorder. Her medical report included classical grade 2 LIS. Her neuropathology report confirmed the marked absence of sulci and further demonstrated a severe degree of pachygyria, with a distinct gray-white demarcation, and the thickness of the cerebral cortex was up to 18–20 mm. In addition, there were areas of apparent gray matter heterotopia presenting. The basal ganglia, including the caudate, putamen, and globus pallidus, were demonstrated grossly with roughly normal size and relationship to each other. The thalamus was also present, and hypothalamic structures were identified. She had abnormalities typical to both Johanson–Blizzard syndrome (JBS) and Miller–Dieker syndrome (MDS). However, the medical records indicated that the mechanism that has led to two different genetic diseases was unknown, yet a relationship between the two could be possible. Her results confirmed a deletion of the LIS critical region on chromosome 17p13.3 based on the deletion of FISH probes L-1232 and 8–1. The postmortem interval was 2 h. 

#### 2.2.2. Control

Case 4, #877, is the control for Case 1 and is the brain tissue of an 8 years and 7-month-old African American female whose cause of death was intracerebral hemorrhage due to an accident at the playground. The postmortem interval was 36 h.

Case 5, #4341, is the control for Case 2 and is the brain tissue of a 13 years and 11-month-old Caucasian male whose cause of death was suicide (hanging). He was not taking any medications and was not smoking or using alcohol or drugs. His final neuropathological diagnosis included acute brain edema and agonal neuronal changes. The postmortem interval was 16 h.

Case 6, #4638, is the other control for Case 2, from a 15 years and one-month-old Caucasian female whose cause of death is chest injuries due to a motor vehicle accident. The postmortem interval was 5 h.

Case 7, #4455, is the control for Case 3, from a 1 years and 3-month-old Caucasian female whose cause of death is drowning. The postmortem interval was 39 h. 

Table 1 shows the clinical background and preservation of brain tissue after death in both groups.

## 3. Results

**Figure 1:**
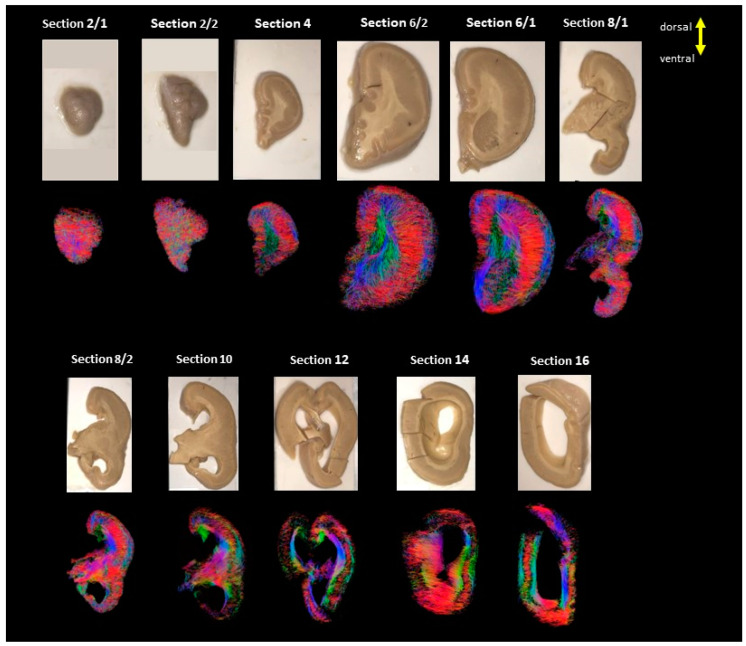
Showing all the sections from LIS, #683, 8 years and 10 months old female. The color-coding of tractography pathways was based on a standard red-green-blue (RGB) code that was applied to the vector in each brain area to show the spatial locations of every segment of each pathway (red for right-left, blue for superior-inferior, and green for anterior-posterior). Composite colors e.g., orange, yellow, purple, and violet indicate combined RGB orientations.

**Figure 2:**
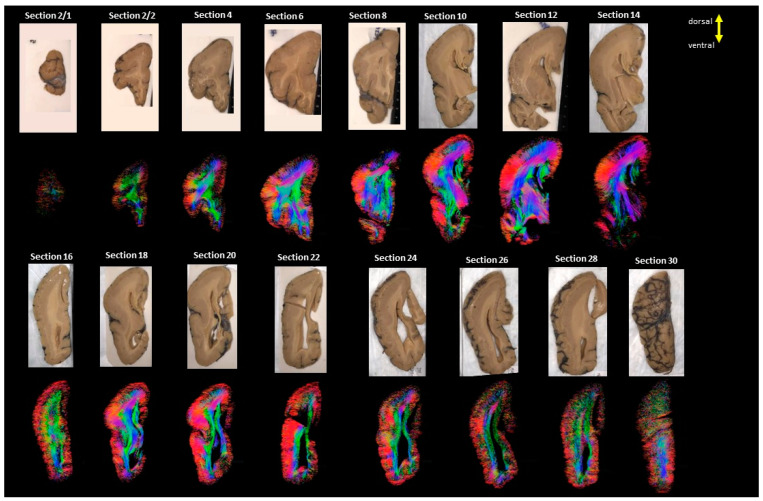
Showing all the sections from LIS, #4524, 13 years and 11 months old male. The color coding of tractography pathways is the same as in Figure 1.

**Figure 3:**
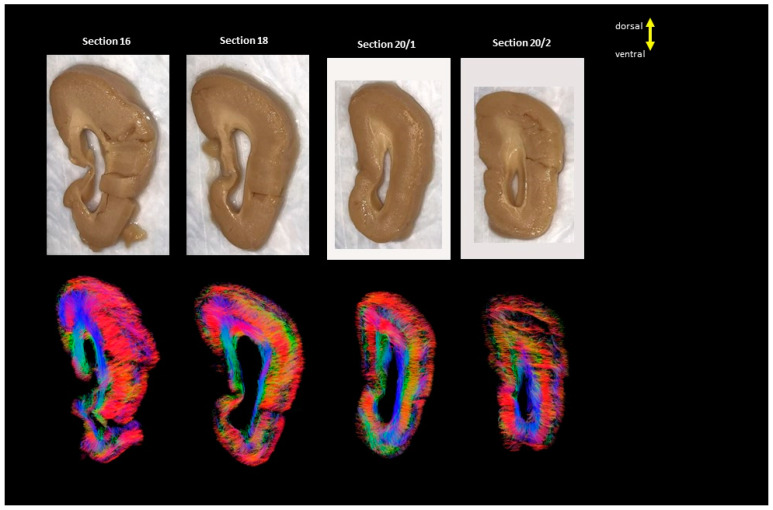
Showing all the sections from LIS, #641, 1 years and 5 months old female. The color coding of tractography pathways is the same as in Figure 1.

**Figure 4:**
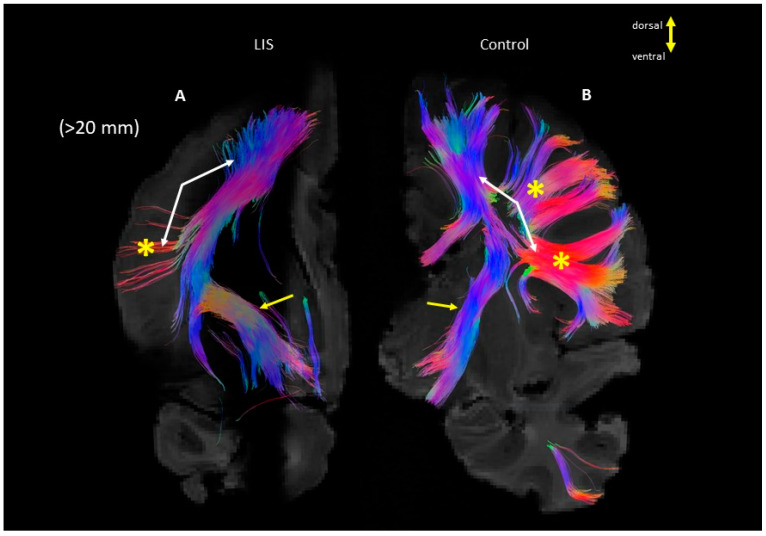
Long tractography pathways (>20 mm) from a section through the hippocampus. (**A**): LIS, (**B**): Control subjects, arrows indicate the projection and the stars and white arrows indicate the long cortico-cortical pathways. (**Left**: LIS #4524, **Right**: control #4341). The color coding of tractography: blue indicates the superior-inferior and red indicates the right-left.

Serial images of the whole hemicoronal specimens of the three LIS patients are shown in Figure 1, Figure 2 and Figure 3. In Case 1 (#683, LIS, 8 years 10 months, female), coronal sections were obtained from almost half of the hemisphere from the anterior to midline (Figure 1). Case 2 (#4524, LIS, 13 years 11 months, male) yielded complete coronal sections from the whole hemisphere (Figure 2). Subcortical fibers were similar to the other two LIS subjects, but long fibers and subcortical gray matter structures were more prominent in this subject (Figure 2 and Figure 4). Unfortunately, Case 3 (subject #641, LIS, 1 year 5 months, female) had only four coronal slabs corresponding to the mid-posterior part of the brain, including significantly enlarged lateral ventricles (Figure 3). 

Identification of long tractography pathways (>20 mm) from a coronal section at the level of the hippocampal level revealed a similar orientation of projection fibers (blue) through the crus cerebri and internal capsule (Figure 4; yellow arrows). In contrast, long (>20 mm) pathways associated with corticocortical fibers in the section were absent in the LIS subject (Figure 4; yellow asterisk and white arrows). Short u-fibers were distinct and thicker in quantities in the control subject (yellow asterisk).

**Figure 5:**
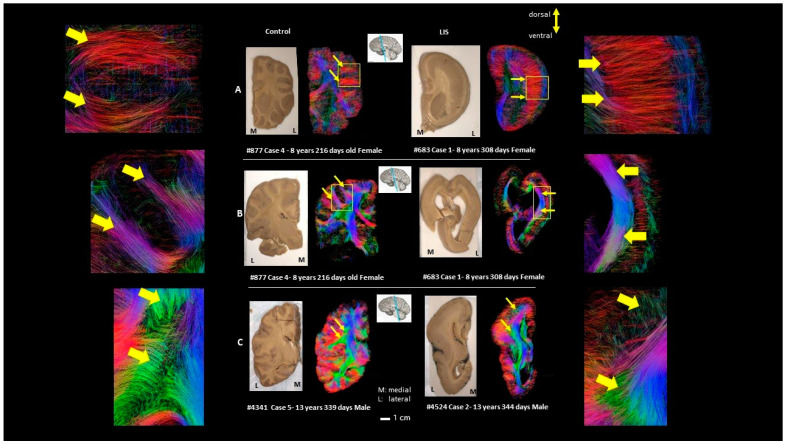
Cortical layer-like abnormalities seen in the comparative qualitative analysis. The color coding of tractography pathways is the same as in Figure 1. (**A**–**C**) show coronal sections through different levels at the optic chiasm, tegmentum of the midbrain, and the isthmus of corpus callosum, respectively. Arrows and squares indicate the typical and corresponding abnormal fibers.

**Figure 6:**
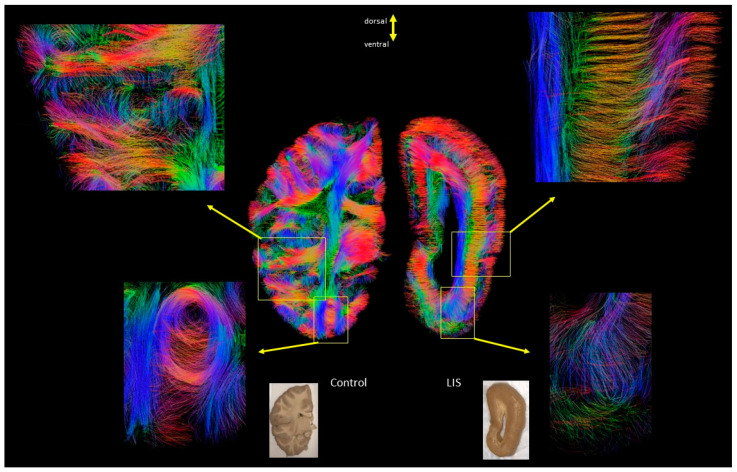
Enlarged sections from the cortical layer-like abnormalities in LIS patients and the corresponding view in healthy controls. (**Left**: control #4555, **Right**: LIS #641). The magnified regions show the typical (left) and abnormal (right) formation of short-range association fibers (upper panels) and fiber organization (lower panels), respectively.

Figure 5, row A, shows the coronal sections through the optic chiasm; row B shows sections through the tegmentum of the midbrain of Case 4 subject #877 (LIS) and Case 1 subject #683 (control); row C shows a coronal section through the isthmus of the corpus callosum of Case 5 subject #4341 (LIS) and Case 2 subject #4524 (control). Abnormalities in the cortical layer-like structures and short u-fibers were magnified in rows A and B. Arrows in row C and the magnified area in the corresponding image show that fibers belonging to the association pathway are reduced and disorganized in the LIS subject compared to the control. Not only association fibers (arrows in Figure 5C) but also radially oriented subcortical fibers (arrows in Figure 5A and circles in Figure 5B) likely associated with residuals of radial neuronal migration pathways and/or u-fibers were reduced in the LIS subjects compared to neurotypical subjects (Figure 5). Magnified views of the area from the edges of the temporal horn of the lateral ventricle to the corresponding cortical area clearly show the same pattern across subjects (Figure 6). 

**Figure 7:**
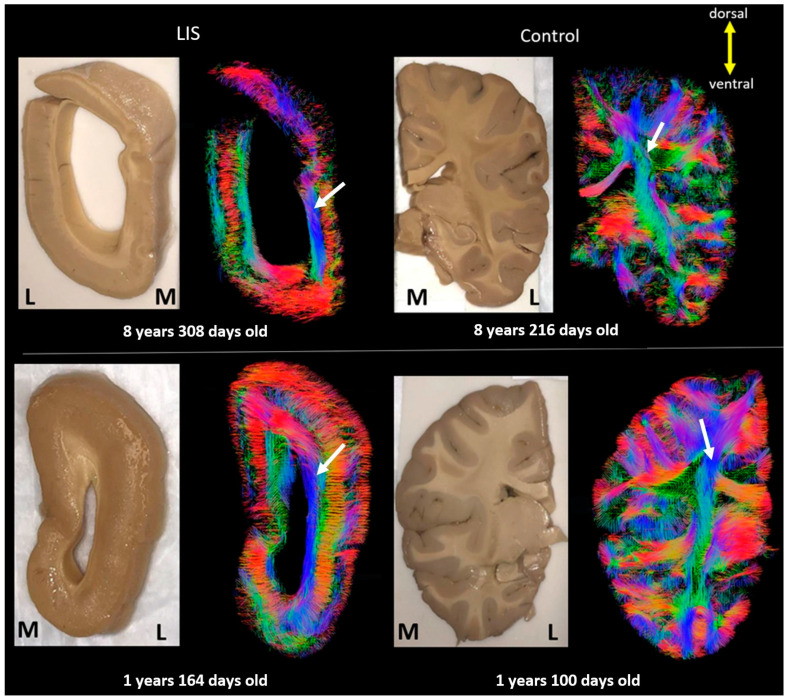
Coronal section through the hippocampal level in infant and pediatric subjects showing severely abnormally developed structures neighboring the ventricles. (**Upper** row: Left: LIS #683, **Right**: control #877; **Lower** row: **Left**: LIS #641, **Right**: control #4455). The color coding of tractography pathways is the same as in Figure 1.

**Figure 8:**
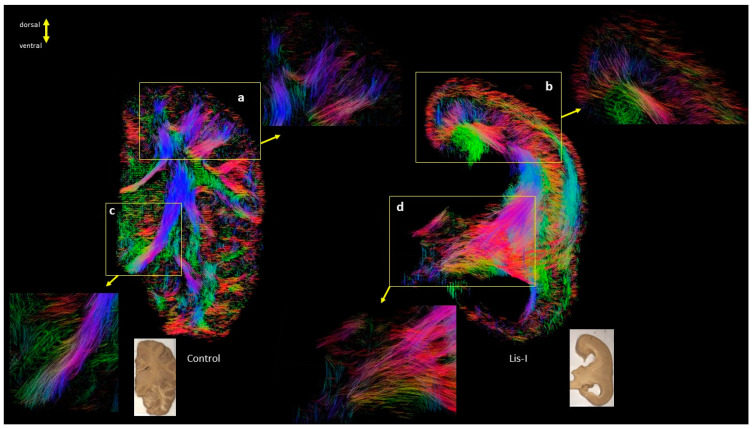
Enlarged sections from the cortical layer-like and white matter projection abnormalities in LIS patients and the corresponding view in healthy controls. (**Left**: control #877, **Right**: LIS #683) (**a**) Enlarged view of the cortex in the healthy control, (**b**) corresponding view of the (**a**) in LIS patient, (**c**) projection fibers passing through the internal capsule in the healthy control, (**d**) corresponding view of the (**c**) in LIS patient. Note that all white matter fibers merge as one single bundle in LIS patients due to agenesis of the structures. This patient was also reported to have agenesis of the corpus callosum. Whole coronal sections and regionally enlarged views (**a**–**d**, see panels pointed with arrows) of the cortical layer-like and white matter projection abnormalities in a LIS patient and a healthy control (**Left**: control #877, **Right**: LIS #683). The color coding of tractography pathways is the same as in Figure 1.

However, in the relatively severe cases in Figure 7 (#683 and #641), the layer-like structures are more pronounced through the cortex and white matter (Figure 7, white arrows), through sections at a level similar to Figure 4 (hippocampus), and with excessively ventricular enlargement in both infant and pediatric LIS patients. Figure 8 also shows magnified views of the subcortical and projection pathways. While the projection pathways through the internal capsule were clearly visible in the control subject (Figure 8c), in the same region of the LIS brain, a bundle of pathways that projected over a wider area than in controls was observed (Figure 8d).

**Figure 9:**
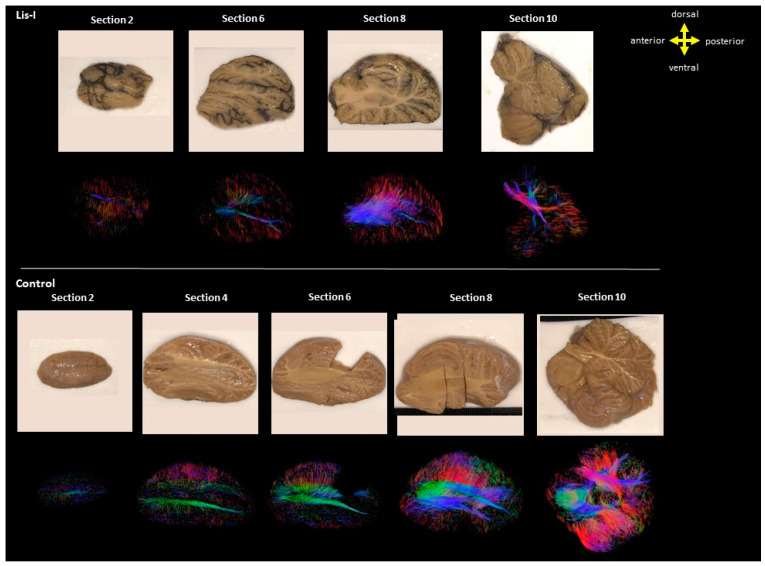
Showing the age-sex matched sections from the lateral surface through the mid-sagittal of the cerebellum. **Upper** row: LIS patient #4524, 13 years and 11 months old male, reported to have hypoplastic degenerating cerebellum. **Lower** row: Control, #4341, 13 years and 11 months old male. The color-coding of tractography pathways for the cerebellum is red for superior-inferior, blue for anterior-posterior, and green for left-right. Composite colors e.g., orange, yellow, purple, and violet indicate combined RGB orientations.

**Figure 10:**
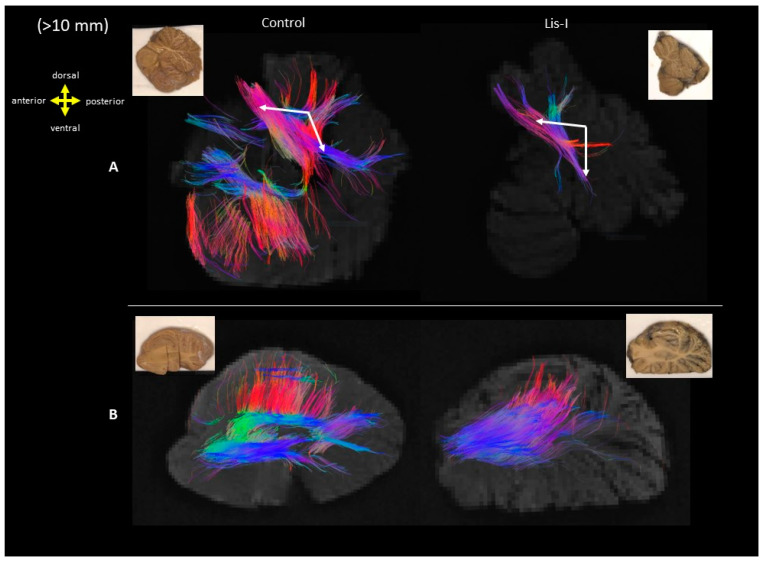
Long tractography pathways (>10 mm) from sections through row (**A**) mid-sagittal and row (**B**) middle cerebellar peduncle. Row (**A**): Arrows show the fibers passing through the superior cerebellar peduncle. (**Left**: control #4341, **Right**: LIS #4524) Row (**B**): Fibers passing through the middle cerebellar peduncle merge as one single bundle in LIS. (**Left**: control #4341, **Right**: LIS #4524). The color coding of tractography pathways is the same as used in Figure 9.

Cerebellar specimens were obtained from only one subject (#4524) and a corresponding age and sex-matched control subject (#4341). Serial slabs of these specimens are shown in Figure 9; the LIS patient was diagnosed with a hypoplastic degenerating cerebellum. Long cerebellar pathways (>10 mm) through the superior cerebellar peduncle were similar in the LIS compared to the control but smaller in volume (Figure 10, row A). However, fibers from the cerebellar cortical areas were almost all missing in the midsagittal section (Figure 10, row B). The long fibers through the section of the middle cerebellar peduncle revealed a similar orientation as the cerebral projection fibers in Figure 5, but the fibers merged as a single bundle (Figure 10, row B), 

## 4. Discussion

Regional white matter pathways were qualitatively analyzed using the diffusion MRI of brain samples from LIS patients and age and sex-matched neurologically healthy subjects. Major anatomical structures were determined, and associated pathways were assessed. The results showed that all LIS brain specimens were disorganized with reduced amounts of association fibers and radially oriented subcortical fibers, likely associated with radial migration pathways and u-fibers. On the other hand, depending on the severity of the case, projection fibers in LIS were similar to those in the controls. These results suggest that patients with LIS exhibit a wide range of abnormalities, including cortical layer-like abnormalities and abnormal connections and orientations of subcortical migration and white matter pathways. 

### 4.1. Developmental Aspect of the LIS Brains

Mutations in lissencephaly 1 (LIS1) and doublecortin (DCX) were the first two genes found to be associated with type I LIS. With advances in molecular genetic analysis, more than 20 LIS/SBH-related genes have been identified, and the number of newly associated genes continues to grow [27]. In addition to impaired neuronal migration processes, disturbed neurogenesis may also be involved in the smooth brain [28]. The failure of postmitotic neurons to reach and properly settle in the final destination in the cortical plate may result in abnormal cortical thickness and reduced or absent gyri and sulci on the cortical surface [28]. This is commonly associated with disorders of axonal elongation and guidance, such as agenesis of the corpus callosum [29]. Deletions of 17p13.3 result in two well-characterized disorders: isolated LIS sequence (ILS) and Miller–Dieker syndrome (MDS). ILS is a diverse disorder characterized by varying degrees of LIS and the absence of other significant abnormalities, such as craniofacial dysmorphism [28]. MDS is distinguished by more severe LIS than ILS, characteristic facial defects (high forehead, short nose with anteverted nares, thin vermilion border, and micrognathia), and, on rare occasions, other deformities [30]. Children with ILS and MDS have marked retardation and epilepsy [31].

In addition, using gene-phenotype links, LIS can be subdivided into seven subtypes: (i) LIS with diffuse agyria and cerebellar hypoplasia, (ii) micro-LIS (combination of LIS and severe congenital microcephaly) with cerebellar hypoplasia, (iii) classic thick LIS, (iv) tubulinopathy-related dysgyria (a mixture of small and large gyri separated by shallow sulci), (v) subcortical band heterotopia, (vi) thin undulating LIS, and (vii) micro-LIS other than tubulinopathies [6]. 

Symptoms encountered in LIS include seizures, intellectual disability, developmental delays, poor motor function, feeding difficulties, and swelling of the extremities [29].

A comparison of the clinical outcome and the neurological features of our cases with the classical subtype of LIS is shown in Table 2. All our patients suffered from various symptoms depending on the grade/severity of LIS.

### 4.2. Microanatomy of Lissencephaly

Patients with LIS exhibit similar abnormalities in neuronal migration and cortical laminar organization. However, the microstructural features of LIS greatly vary [32], along with the severity in the neocortex and the extent of lesions in the affected regions [33]. Consistent with this, our three subjects had different levels of affection and common abnormalities (Table 2).

The disorganized fibers of the association pathways seen in our specimens might be a good example of histopathological findings in the white matter of LIS, where heterotopic neurons are scattered, and axons are randomly oriented due to axonal guidance defects [34]. Abnormalities of white matter tracts have been previously reported [2,27], as well as hypoplasia, disorganization, or the complete absence of the corticospinal tract (CST) in patients with LIS [35,36,37,38].

The advantages of using DTI to study the anatomy of CST have been highlighted in the identification of abnormalities and phenotypic variabilities in various brain disorders, including LIS and gyral disorganization with dysplasia of the basal ganglia and corpus callosum [39]. Our previous study reported the proper directions and locations of projection fibers in both types of patients (LIS1 and DCX mutations) [40]. Similarly, in one of our subjects (#4524 Case 2, Figure 4), we were able to analyze similar tracts at the section level in the LIS and control subjects. However, in another subject in this study, fibers passing through the internal capsule were integrated with adjacent fibers as one large bundle approximately at the level of the mammillary body (#683 Case 1, Figure 8). This could be a notable example of how the severity of LIS affects white matter organization. The LIS subjects shown in Figure 4 and Figure 8 highlight distinct variations in the organization of projection fibers passing through the internal capsule. For instance, a moderately affected grade 3 subject (#4524 Case 2, Figure 4) exhibits well-defined and structured fibers, similar to the control group, while a severely affected subject (#683 Case 1, Figure 8) shows disorganized and dispersed fibers.

LIS with cerebellar hypoplasia (LCH) is the term used to describe both classical and non-classical forms of LIS that are linked to the underdevelopment of the cerebellum. This distinctive category of brain malformations has been recognized only relatively recently [41]. The subtypes of the LCH are not well defined [41]. Defects in proteins expressed by the TUBA1A, RELN, and very low-density lipoprotein receptor (VLDLR) genes have been documented in patients with substantial cerebellar abnormalities [42]. In our study, the cerebellum of LIS subject #4524 (Case 2) had hypoplastic degeneration with a relatively low number of fibers (Figure 9 and Figure 10). The low number of fibers and the misorientation of those fibers paralleled the diagnosis. The same subject also had heterotopic remnants of the inferior olive. The inferior olive supplies climbing fibers to Purkinje cells in the cerebellar cortex [43], which project mainly through the inferior cerebellar peduncle to the cerebellar cortex and deep cerebellar nuclei. Thus, the formation of the olivocerebellar loops may have been affected. However, the inferior cerebellar peduncle could not be identified in the sagittal sections of the cerebellum. Analysis of the longer fibers also indicated that fibers through the regions where the deep cerebellar nuclei reside were integrated as a single pathway in LIS, while distinctive fiber bundles were present in the control subject (Figure 10).

### 4.3. Limitations and Future Directions

Because LIS is a rare disease, the sample size was small, and only qualitative analysis was performed in this study. In addition, because we used brain slabs instead of whole hemispheres or brains, we were unable to analyze the starting/ending regions of long-range pathways running in the anterior-posterior direction. However, our high-resolution ex vivo MRI was successful in finding common abnormalities (e.g., abnormalities in the radial pathway) across the samples. Further analysis using optic imaging and histology will reveal the neurological correlates of this finding. In addition, for future studies, it would be comprehensive to compare the degree of neuronal migration by tractography between type I LIS (under migration) and type II LIS (over migration).

**Table 2:** Comparison of our cases with the summary of the clinical and neuroradiological features of classical lissencephaly subtypes *.

	LIS1	ARX	DCX	TUBA1A	RELN	VLDLR	Our Case 1 #683	Our Case 2 #4524	Our Case 3 #641
Chromosome location	17p13.3	Xp22.13	Xq22.3-q23	12q13.12	7q22.1	9p24.2	17p13.3	17p13 (submicroscopic deletion)	17p13.3
Head size/circumference	Relative/acquired microcephaly	Microcephaly	Relative/acquired microcephaly	Microcephaly	Normal or acquired mild microcephaly	Normal	Microcephaly with tririgonocephaly	Dysmorphism	Microcephaly
Early-onset seizures	At few months of age	Present	At few months of age	Present	At few months of life	Present (uncommon)	No seizures recognized	N/A	Present
Ophthalmologic findings reported	N/A	Duane anomaly (rare)	Nystagmus	Strabismus, nystagmus	Myopia, strabismus, nystagmus	Strabismus, nystagmus, cataracts (rare)	Bilateral micropthalmia with microcornea and complete colobomas bilateratelly. (Considered blind)	N/A	Bilateral disk hypoplasia (pupils were bilaterally unreactive)
Brain MRI features:									
Optic nerves	N/A	Hypoplasia (rare)	N/A	Hypoplasia (rare)	Normal or atrophy	N/A	Present	Present	Hypoplasia
Cerebellum	Normal or mild vermis hypoplasia	Normal or hypoplastic/dysplastic	Normal or cerebellar vermis hypoplasia (could be worse than that seen in LIS1)	Normal or mild to severe cerebellar vermis > hemispheres hypoplasia	Normal to profound cerebellar hypoplasia, especially inferior vermis and hemispheres, afoliation	Inferior cerebellar hemispheres and vermis hypoplasia, afoliation	The cerebellar vermis slightly small and is incorporated in the cyst involving the hilum of the left cerebellar hemisphere. The dentate nucleus is indistinct.	Severe cerebellar atrophy/hypoplasia	Atrophy of the cerebellar vermis and small peduncles. But no atrophy at cerebellar hemispheres
Brainstem	Normal or hypoplastic	Mild hypoplasia	Normal or uncommonly hypoplastic	Mild to severe hypoplasia	Hypoplasia	Hypoplasia particularly the pons	No significant anomalies	Heterotopic remnants of inferior olive in the mid medulla	Normal
Cerebral cortex gyration	Moderate agyria in occipital lobes transitioning to pachygyria anteriorly	Posterior agyria and anterior pachygyria	Agyria more prominent in anterior regions and pachygyria posteriorly	Agyria/pachygyria, perisylvian polymicrogyria or pachygyria	Moderate pachygyria	Normal to mild simplification, predominantly frontal pachygyria	Severe agyria. Only inferior frontal sulci and major fissures seen.	Pachgyria from frontal lobe to Slyvian fissure, agyria in the dorsal brain.	Severe degree of pachgyria. Failure of formation of the usual gryal and sulcal definition (agyria) leaving only a vague suggestion of thr Slyvian sulcus and central sulcus as well as the parieto-occipital sulcus.
Gradient of gyration abnormality	p > a	p > a	p < a	p > a	p < a	None or variable (p < a more common)	N/A	p > a	N/A
Cerebral cortical thickness	Abnormally thick cortex	Moderately thick cortex	Thick cortex, SBH in female carriers	Thick cortex or SBH/SBH-like	Thick cortex	Normal to mildly thick cortex	Abnormally thick cortex	Thick (in excess of 1.5–2 cm) and has abnormal organization.	Abnormally thick cortex up to 18–20 mm (3–4 times normal thickness)
Hippocampus	N/A	N/A	N/A	Small and globular	Flat and lacking definable upper and lower blades	Normal	N/A	Hypoplastic	N/A
Basal ganglia/thalami	N/A	Dysplastic	N/A	Very small and dysplastic	N/A	N/A	Abnormally disposed	Normal	Normal
Internal capsule	N/A	N/A	N/A	Absent or hypoplastic anterior limbs	N/A	N/A	N/A	N/A	N/A
Corpus callosum	Could be hypoplastic	Absent	Normal, mild hypoplasia or absent	Dysmorphic, thin or absent	Hypoplastic	Normal	Formed, thin	Only small remnant	Clearly formed
Lateral ventricles	Enlarged	Enlarged	Enlarged	Mildly to severely enlarged	Enlarged	Could be enlarged	Enlarged posteriorly	Enlarged posteriorly	Only the aqueduct was enlarged

* Abbreviations: a, anterior; ARX, aristaless-related homeobox; DCX, doublecortin, LIS 1, type I LIS; N/A, not available; p, posterior, RELN, reelin; SBH, subcortical band heterotropia; TUBA1A, tubulin alpha-1A; VLDLR, very low-density lipoprotein receptor. (The table was adapted with permission from the [42] Copyright 2014 by SAGE Publications).

## 5. Conclusions

Our study focused on analyzing the type I LIS related aberrant neuronal migration and white matter pathways, aiming to provide a comprehensive view of the neuroanatomical characteristics of these changes. Our examination revealed a noticeable increase in radially oriented subcortical fibers that were anatomically associated with radial migration pathways. Furthermore, a noteworthy reduction in association fibers was observed across all type I LIS specimens. Our high-resolution diffusion imaging data will serve as a regional anatomical atlas for type I LIS related nerve fibers, creating a foundation for further comparative studies.

## Figures and Tables

**Table 1:** Clinical background and preservation of brain tissue after death in both groups.

	Brain Age (Years)	Disease/Cause of Death	Sex	Race	Postmortem Interval (Hours)	Time in Fixative (Years) *
Case 1, #683	8	Multiple congenital anomalies	Female	Caucasian	3	24+
Case 2, #4524,	13	Lissencephaly	Male	Caucasian	9	16+
Case 3, #641	1	Lissencephaly	Female	Caucasian	2	24+
Case 4, #877	8	Intracerebral hemorrhage	Female	African American	36	22+
Case 5, #4341	13	Suicide	Male	Caucasian	16	11+
Case 6, #4638	15	Chest Injury/Motor Vehicle Accident	Female	Caucasian	5	15+
Case 7, #4455	1	Drowning	Female	Caucasian	39	3+

* Indicates the time between the time of death (and then subsequent processing of the brain) until it got shipped to our institution. All specimens were processed in the year after they were received.

## Data Availability

The data presented in this study could be available on request. We will evaluate the request case by case, consulting with the Brain Bank.
